# Exploring the Relationship Between Halide Substitution, Structural Disorder, and Lithium Distribution in Lithium Argyrodites (Li_6–*x*_PS_5–*x*_Br_1+*x*_)

**DOI:** 10.1021/acs.chemmater.3c01525

**Published:** 2023-09-18

**Authors:** Ajay Gautam, Hanan Al-Kutubi, Theodosios Famprikis, Swapna Ganapathy, Marnix Wagemaker

**Affiliations:** Storage of Electrochemical Energy, Department of Radiation Science and Technology, Faculty of Applied Sciences, Delft University of Technology, Mekelweg 15, 2629 JB Delft, The Netherlands

## Abstract

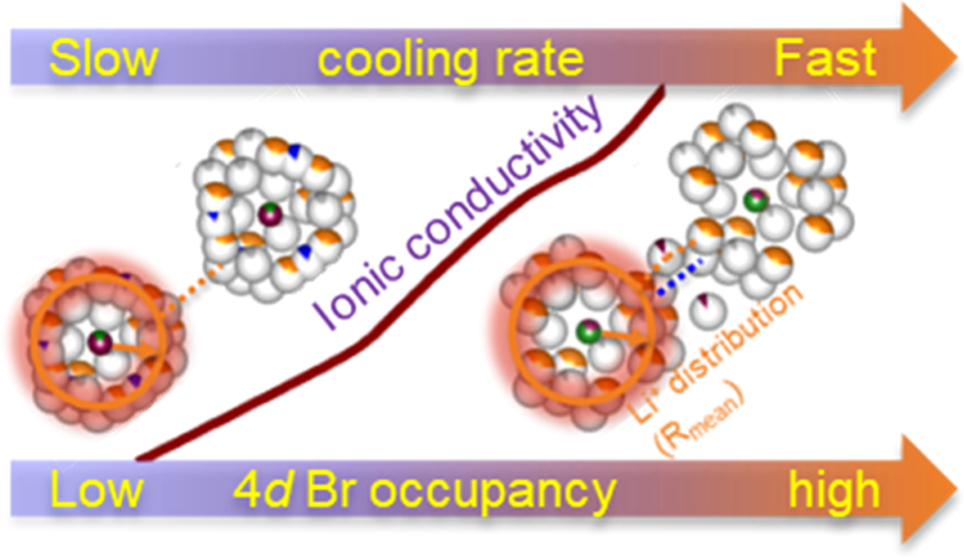

Lithium argyrodite superionic conductors have recently gained significant attention as potential solid electrolytes for all-solid-state batteries because of their high ionic conductivity and ease of processing. Promising aspects of these materials are the ability to introduce halides (Li_6–*x*_PS_5–*x*_Hal_1+*x*_, Hal = Cl and Br) into the crystal structure, which can greatly impact the lithium distribution over the wide range of accessible sites and the structural disorder between the S^2–^ and Hal^–^ anion on the Wyckoff 4*d* site, both of which strongly influence the ionic conductivity. However, the complex relationship among halide substitution, structural disorder, and lithium distribution is not fully understood, impeding optimal material design. In this study, we investigate the effect of bromide substitution on lithium argyrodite (Li_6–*x*_PS_5–*x*_Br_1+*x*_, in the range 0.0 ≤ *x* ≤ 0.5) and engineer structural disorder by changing the synthesis protocol. We reveal the correlation between the lithium substructure and ionic transport using neutron diffraction, solid-state nuclear magnetic resonance (NMR) spectroscopy, and electrochemical impedance spectroscopy. We find that a higher ionic conductivity is correlated with a lower average negative charge on the 4*d* site, located in the center of the Li^+^ “cage”, as a result of the partial replacement of S^2–^ by Br^–^. This leads to weaker interactions within the Li^+^ “cage”, promoting Li-ion diffusivity across the unit cell. We also identify an additional T4 Li^+^ site, which enables an alternative jump route (T5–T4–T5) with a lower migration energy barrier. The resulting expansion of the Li^+^ cages and increased connections between cages lead to a maximum ionic conductivity of 8.55 mS/cm for quenched Li_5.5_PS_4.5_Br_1.5_ having the highest degree of structural disorder, an 11-fold improvement compared to slow-cooled Li_6_PS_5_Br having the lowest degree of structural disorder. Thereby, this work advances the understanding of the structure–transport correlations in lithium argyrodites, specifically how structural disorder and halide substitution impact the lithium substructure and transport properties and how this can be realized effectively through the synthesis method and tuning of the composition.

## Introduction

Lithium-ion batteries are the leading battery technology used in portable electronics and electric vehicles, demonstrating constant enhancement/improvement in electrochemical performance resulting from both materials and engineering development.^1−3^ However, lithium-ion batteries utilize organic liquid electrolytes with a high flammability risk and limited energy density due to their incompatibility with the lithium metal anode.^4,5^ All-solid-state batteries (ASSBs) replace liquid electrolytes with solid electrolytes and may enable the use of a lithium/silicon metal anode, which could offer a higher energy density and improved safety.^5−9^ A crucial component of ASSBs is an ionically conductive solid electrolyte that can provide stable interfaces with the positive and negative electrodes.^9^ To achieve this, various solid electrolytes have been developed so far including oxides,^10−12^ phosphates,^13^ lithium halides,^14−20^ and lithium (halide-enriched) argyrodites (Li_6–*x*_PS_5–*x*_Hal_1+*x*_, Hal = Cl and Br).^21−26^ Among all of them, lithium argyrodites have gained relatively large interest because of their higher ionic conductivity, favorable mechanical properties, and ease of processing.^27,28^ High conductivities are required for fast charging and high power applications and generally allow more efficient use of the energy stored in the battery. Further, improving conductivity and developing effective synthesis routes demand a better understanding of the conduction mechanism and its correlation to the structure.

**Figure 1:**
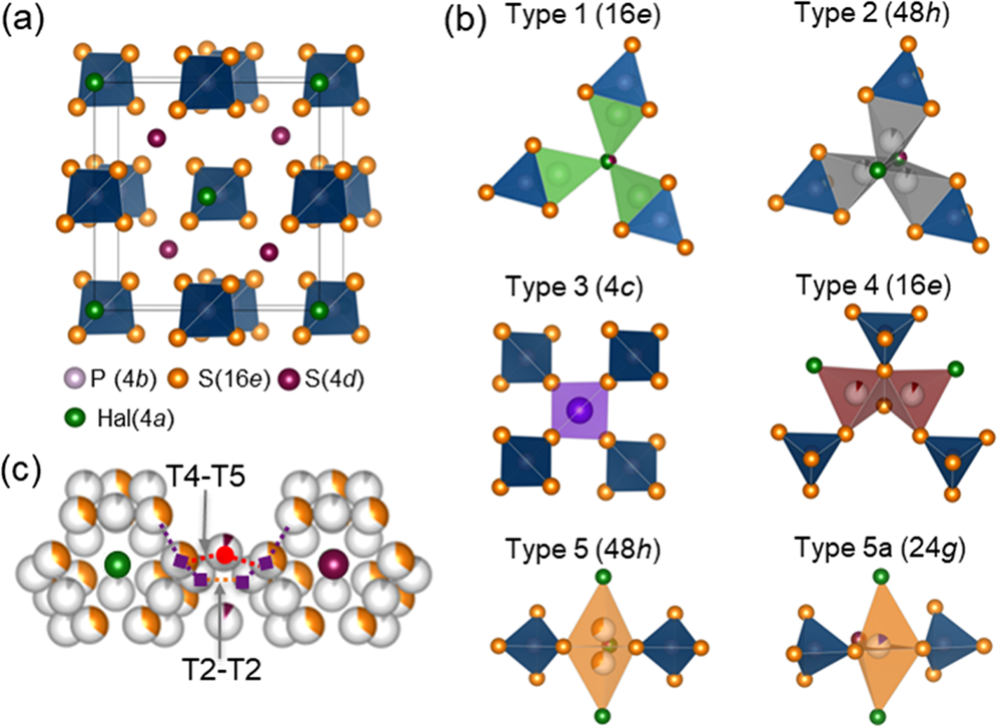
(a) Crystal structure of Li_6–*x*_PS_5–*x*_Hal_1+*x*_ (Hal = Cl, Br, I), with an ordered state. Hal^–^ occupies the Wyckoff 4*a* site and S^2–^ occupies the Wyckoff 4*d* site. The remaining S^2–^ occupies the Wyckoff 16*e* site. The argyrodite framework forms 136 interstitial tetrahedral voids, of which 4 are occupied by P^5+^, forming PS_4_^3–^. Lithium is removed from the unit cell. (b) Panels showing the trigonally coordinated type 5*a* site and the five tetrahedral interstitial sites (types T1–T5) that can accommodate lithium ions (proposed in ref (34)). (c) Lithium (T5, T2, T5a, and T4 sites) forms the cages around the S^2–^/Hal^–^ anions (4*d* site); T2–T2 and T5–T4–T5 offer the shortest intercage jump distance (orange dotted line and red dotted line, respectively).

The halide-based argyrodite family is considered one of the most promising classes of solid electrolytes exhibiting very high ionic conductivities at room temperature.^26^Figure 1a shows the ideal crystal structure of (Li_6_PS_5_Hal) argyrodite in the ordered state having the cubic *F*4̅3*m* space group. In the face-centered cubic lattice, halide ions occupy the tetrahedral Wyckoff 4*a* sites and S^2–^ not bonded to P occupies half of the tetrahedral sites (Wyckoff 4*d*). The argyrodite cubic phase anion framework forms 136 interstitial tetrahedral voids per unit cell available for cation occupancy.^11,29^ Four voids are occupied by P^5+^ cations on the Wyckoff 4b site, forming PS_4_^3*–*^ tetrahedra, together with S on the Wyckoff 16*e* site. The remaining 132 voids can be occupied by the lithium sites and categorized according to how many S ions (16*e*) are shared (face, edge, corner) with the PS_4_^3*–*^ tetrahedra.^11,30^ Thus, the tetrahedral site can be split into five distinct tetrahedral types (types 1–5), as shown in Figure 1b, that were described by Deiseroth et al.^29,31,32^ The type 1 (T1) and type 2 (T2) sites share faces and edges with PS_4_^3*–*^. The type 3 (T3) and type 4 (T4) tetrahedral sites share 4 and 3 corners with PS_4_^3*–*^, respectively.^11,29,31−33^ The type 5 (T5) sites share 2 corners with PS_4_^3–^ and the type 5a (T5a) sites lie in between the faces of 2 neighboring T5 sites in a trigonal bipyramidal environment^11^ because of their close proximity, as shown in Figure 1b. The T5, T2, T4, and T5a sites form a cage-like substructure around the free sulfur anions (4*d* site), as shown in Figure 1c, which form the 3D Li^+^ network throughout the structure.

De Klerk et al.^35^ proposed three different jump processes for lithium-ion conduction pathways: intracage and doublet jumps (within the cage) and intercage jump (between the cages).^35^ In these DFT simulations, T5 and T5a Li sites were considered, whereas a recent study has suggested additional lithium sites to be involved that impact the diffusion pathway and mechanism.^36,37^ Minafra et al. recently reinvestigated the lithium argyrodite substructure for Li_6_PS_5_Hal (Hal = Cl, Br, I), revealing additional T2 lithium occupancies and suggesting new lithium conduction pathways.^37^ In other sulfide argyrodite compositions, a partial occupancy of additional (T2, T3, or T4) lithium sites was discovered.^11,38−40^ For instance, the partial substitution of Ge^4+^ in the P^5+^ site in Li_6+*x*_P_1–*x*_Ge_*x*_S_5_I found a partial occupancy of T2 and T4 sites,^41^ which provides shorter and favorable paths for Li^+^ diffusion, thereby increasing the ionic conductivity from 10^–3^ to ∼10 mS/cm.^41^ Another argyrodite series, the Sn-substituted thioantimonate argyrodites, exhibited T2 and T3 site occupancies, and Li_6.15_Al_0.15_Si_1.35_S_5.4_O_0.6_^42^ exhibited T4 site occupancies.^42^

Anion site disorder refers to the random arrangement of various anions within the same crystallographic site (in this case, the Br occupancy on the 4*d* site).^43^ The iodide version represents the ordered arrangement where the 4*a* site is fully occupied by I^*–*^ (I^*–*^ (Wyckoff 4*a*) = 1), while the 4*d* site is exclusively occupied by S^2–^, a result of the large difference in ionic radii. In this case, poor ion diffusion between the Li-ion cages presents the rate-limiting step for long-range diffusion.^35^ Following the decreasing difference in ionic radii, the site disorder increases for bromide, followed by the chloride version having the highest site disorder of S^2*–*^/Hal^*–*^ on the 4*d* site (Br^–^ (4*d*) = 20%, Cl^*–*^ (4*d*) = 60%).^43^ DFT simulations predict that the maximum conductivity may be achieved with around 75% site disorder, where the jump rate frequency of the three jump processes, doublet, intra-, and intercage, is similar.^35^ Halide substitution is also shown to increase conductivity. Adeli et al.^26^ investigated the higher chloride substitution in Li_6–*x*_PS_5–*x*_Cl_1+*x*_ (*x* ≤ 0.5) with a conductivity of 9 mS/cm achieved at *x* = 0.5. In addition to different halides, Yu et al.^44^ reported an ionic conductivity of 4.13 mS/cm for the higher bromide-substituted Li_6–*x*_PS_5–*x*_Br_1+*x*_ (*x* ≤ 0.5).^44^ Despite these significant advances, the relationships between long-range diffusion and structure, especially with regard to sublattice disorder, are still not well understood.

In this study, we investigate the effect of halide substitution (Li_6–*x*_PS_5–*x*_Br_1+*x*_, in the range 0.0 ≤ *x* ≤ 0.5) on lithium argyrodite and engineer structural disorder by changing the synthesis protocol. First, an argyrodite precursor was prepared via mechanical milling, followed by heat treatment at a particular temperature to obtain crystalline argyrodite. Two different cooling methods were employed to influence the extent of structural disorder. Rietveld refinement against the neutron diffraction patterns was performed, demonstrating that this preparation method influences the structural disorder. We found that higher ionic conductivity is correlated with a less negative charge on the 4*d* site as replacing S^2–^ with Br^–^ leads to a lowered average charge on the 4*d* site and weaker interactions within the Li^+^ “cage”, promoting a diffusion pathway for Li^+^ ions across the unit cell. We also identified an additional T4 Li^+^ site in bromide-based argyrodite, which enables an alternative jump route (T5–T4–T5) with a lower migration energy barrier. The resulting expansion of the Li^+^ cages and the increased connections between the cages lead to a maximum ionic conductivity of 8.55 mS/cm. Overall, this work provides a deeper understanding to further improve the ionic conductivity of lithium argyrodites and other solid electrolytes.

## Experimental Section

### Mechanochemical Milling and Postannealing

Li_6–*x*_PS_5–*x*_Br_1+*x*_ syntheses were performed under an argon atmosphere to prevent the contamination of oxygen (O_2_ < 2 ppm) and water (H_2_O < 1 ppm). Li_6–*x*_PS_5–*x*_Br_1+*x*_ was synthesized using mechanochemical milling (Fritsch Pulverisette 7 premium line). The initial precursors, lithium sulfide (Li_2_S, 99.98%), lithium bromide (LiBr, 99.99%), and phosphorus pentasulfide (P_4_S_10_, 99%), were purchased from Merck and Sigma-Aldrich. All precursors were mixed in an appropriate stoichiometric ratio using a mortar and pestle. The obtained 1.0 g of the precursor was then mechanochemically milled (the milling media to precursors ratio is 30:1) using 10 mm-diameter ZrO_2_ balls at 510 rpm for 25 h (every 10 min milling and 10 min rest). After each cycle of milling, the direction of rotation was reversed to achieve better mixing of the precursors. The powder was pressed into a pellet and then placed inside a quartz ampule. Before filling, the quartz ampules were heated in a Buchi at 473 K under vacuum for 12 h to remove traces of water. The quartz ampules were sealed under vacuum (<10^–3^ mbar). The sealed quartz ampules were placed inside a furnace for crystallization, where a ramping rate of 100 K/h was applied to reach a temperature of 550 °C for *x* = 0.0 and 430 °C; for *x* = 0.3 and 0.5 (Li_6–*x*_PS_5–*x*_Br_1+*x*_). After a reaction time of 2 h, two different cooling methods were applied: (1) fast cooling by quenching in liquid nitrogen or (2) slow cooling using a cooling rate of 4 K/h during 5–6 days. The final obtained powder was hand-ground and stored in an argon-atmosphere glovebox. The phase purity, lithium substructure, and ionic transport of the argyrodite series were analyzed by X-ray diffraction, neutron powder diffraction, electrochemical impedance spectroscopy, and NMR spectroscopy.

### X-ray Diffraction

X-ray diffraction was carried out to determine the phase purities and relevant structural parameters with an XˈPert Pro X-ray diffractometer (PANalytical) in Bragg–Brentano θ–θ geometry with Cu Kα radiation (λ_1_ = 1.540598 Å and λ_2_ = 1.544426 Å, at 45 kV and 40 mA). Measurements were taken in the 2θ range between 10 and 90°. All powders were placed in an airtight sample holder with a Kapton lid under an argon atmosphere to prevent air exposure.

### Neutron Powder Diffraction

Neutron powder diffraction data were collected on a PEARL neutron powder diffractometer at the research reactor of the TU Delft,^45^ operating at room temperature and aneutron wavelength of λ = 1.667 Å (selected using the 533 reflections of the germanium monochromator) For each sample, approximately 1.5 g was loaded into a 6 mm-diameter cylindrical vanadium can under an argon atmosphere and then sealed using an indium wire to prevent air exposure during transport. The sample was measured for 6–18 h from 10° < 2θ ≤ 160°, under vacuum. A reference measurement of the empty sample can was subtracted from each of the reported diffractograms.

### Bond Valence Site Energy (BVSE)

BVSE analysis was performed using the softBV software tool with a grid size of 0.1 Å.^46−48^ The crystallographic information file (CIF) obtained from Rietveld refinement of the neutron diffraction data of the Li_6–*x*_PS_5–*x*_Br_1+*x*_ argyrodite series was used to visualize the lithium substructure. An input file (.cube) was generated in softBV, and the BVSE landscape was plotted in VESTA^50^ to visualize the Li-ion migration paths across the unit cell. More details of the BVSE model can be found in refs (47−49).

### Electrochemical Impedance Spectroscopy

Temperature-dependent electrochemical impedance spectroscopy was performed using an Autolab PGSTAT with an EC10 M impedance analyzer using 0.01 V amplitude and frequency ranging from 10 MHz to 1 Hz to determine the ionic conductivity and activation energy of the samples. Approximately 200 mg of powder was pressed into pellets with 10 mm diameter using 300 MPa of pressure for 1 min. Stainless steel disks were attached on both sides of the pellet.^49^ The temperature range was −40 to 10 °C with a step size of 10 °C (Fryka climate chamber). Analyses of the impedance data were performed using RelaxIS 3 impedance spectrum analysis software.

### Rietveld Refinement

TOPAS software^50^ was used to perform Rietveld refinements of X-ray diffraction and neutron diffraction data. As a starting point for this study, the structural information obtained by the neutron powder diffraction of Li_6_PS_5_Br from ref (39) was used. The refinement fits were evaluated using the goodness-of-fit (GOF) fit indicator and *R*_wp_. The following parameters were refined: 15 coefficients for a Chebyshev function were used to fit the background and a modified Thomson–Cox–Hasting pseudo-Voigt function was used for the analyzed peak shape, scale factor, zero error, lattice parameter, isotropic atomic displacement parameter, and atomic occupancies of the free S^2–^ (Wyckoff 4*d*) and Br^–^ (Wyckoff 4*a*) anions since these two anions can be exchanged. The occupancies of Br^–^ and S^2–^ on the Wyckoff 4*a* and Wyckoff 4*d* sites were constrained to 1 (occupancies of Br^–^ (4*a*) + S^2–^ (4*a*) = 1, occupancies of S^2–^ (4*d*) + Br^–^ (4*d*) = 1), assuming full occupancy of these sites by S^2–^ and Br^–^. The stability of the refinements was ensured by allowing the refinement of multiple correlated parameters simultaneously. Finally, lithium occupancies at the possible interstitial sites were determined. All lithium positions and occupancies were fitted during Rietveld refinement where the starting conditions from Gautam et al.^39^ were used as a reference for both the lithium position and occupancy. When a negative value for the thermal displacement parameter or occupancies occurred, the lithium occupancy was assumed to be below the detectable limit and the specific position was not included in the refinement model. The Fourier difference map was calculated to identify visible indications of the Li occupation at particular sites. The overall Li^+^ content was constrained to maintain a charge balance with the refined Br content. The constraints on individual anion/cation occupancies are available in Tables S2–S7.

### Solid-State NMR

Solid-state ^6^Li NMR spectra were obtained on a Bruker Ascend 500 magnet with a static magnetic field *B*_0_ = 11.7 T equipped with a NEO console, corresponding to a ^6^Li Larmor frequency of 73.6 MHz. Sample powders were packed into zirconia rotors of 4 mm diameter, and a MAS speed of 5 kHz was used. The spin–lattice relaxation time (T1) was determined using a saturation-recovery pulse program.^51^ The 90° pulse and T1 were determined for each sample individually. A recycle delay of 5 times the T1 was used for the single-pulse spectra to ensure full relaxation. All spectra were referenced against LiCl in water at 0 ppm.

## Results and Discussion

### Structural Changes Induced by the Br Content and Synthesis Method

Lithium argyrodite Li_6_PS_5_Hal, where Hal^–^ represents Cl, Br, or I, exhibits structural disorder between Hal^–^ and S^2–^ on the 4*d* positions, where higher disorder has been shown to result in higher Li-ion conductivities.^37,44^ Recently, it was discovered that structural disorder can be tuned in single-composition Li_6_PS_5_Br via the synthesis method.^39^ Furthermore, replacing S^2–^ with Br^–^ has been shown to increase the ionic conductivity. However, the effects of this substitution on the lithium substructure and structural disorder are not well understood. In order to fully understand the influence of Br substitutions in lithium argyrodites and the role of the Br^–^/S^2–^ disorder, a mechanochemical method was explored to synthesize Li_6–*x*_PS_5–*x*_Br_1+*x*_ materials (*x* ≤ 0.5), where the initial precursors (Li_2_S, P_4_S_10_, and LiBr) were mechanically milled to ensure mixing, as reported previously,^38^ and subsequently subjected to heat treatment at different temperatures (550 °C for *x* = 0.0 or 430 °C for *x* = 0.3 and 0.5) for 2 h, which was sufficient to achieve crystalline lithium argyrodite. In order to control site disorder (in this case, the 4*d* Br occupancy), two distinct cooling methods were applied: (1) The obtained precursors were slowly cooled at a rate of 4 °C/h over several days to minimize the Br occupancy on the 4*d* site in each composition. (2) The obtained precursors were quenched in liquid nitrogen (fast cooling) to achieve a higher Br occupancy on the 4*d* site. The quenching was expected to “freeze in” the higher Br occupancy on the 4*d* site that was achieved at higher (synthesis) temperatures.

**Figure 2:**
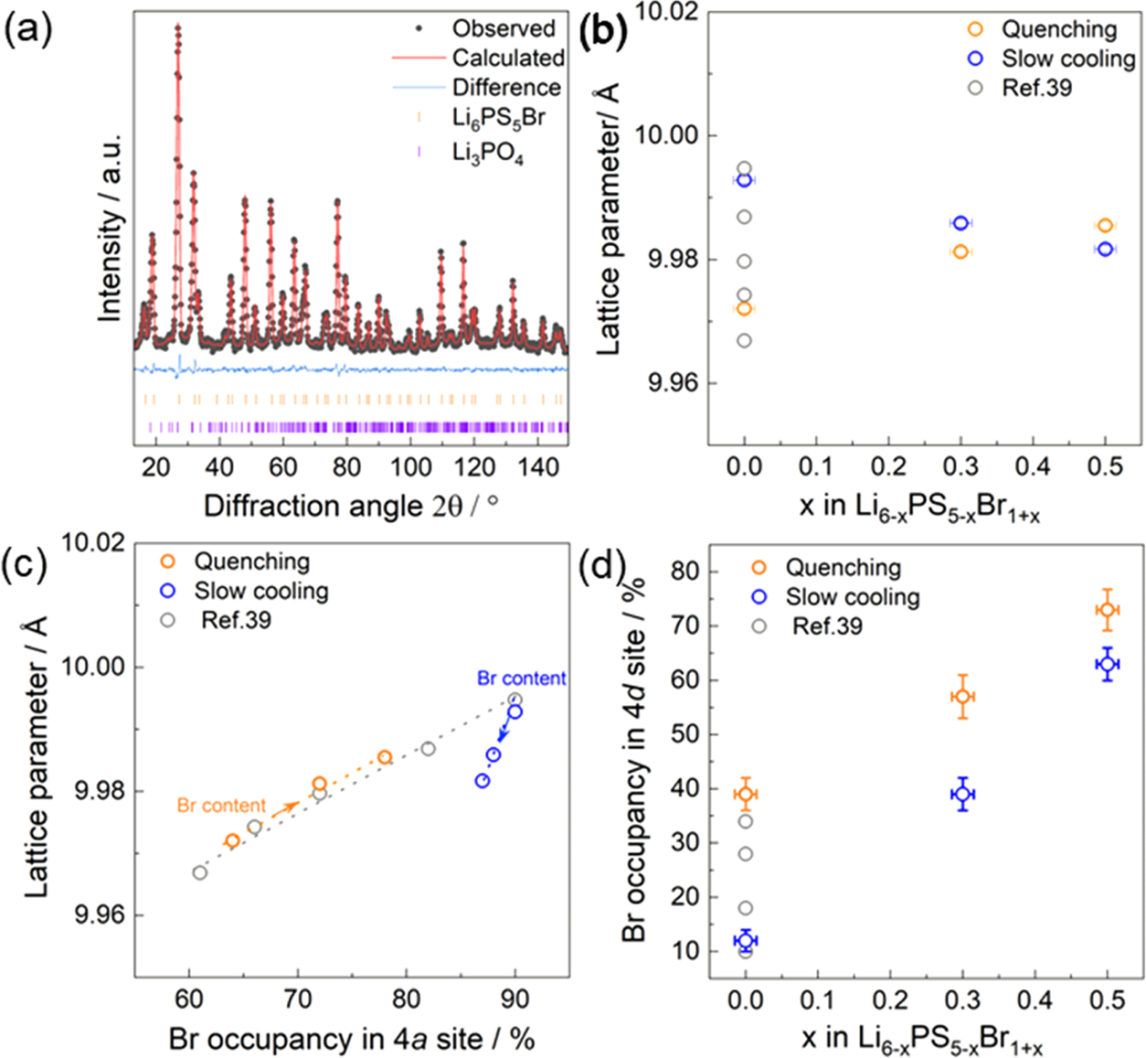
(a) Rietveld refinement analysis of the neutron diffraction data of slow-cooled Li_6_PS_5_Br (*x* = 0), showing a small fraction (0.5 wt %) of the impurity phase of Li_3_PO_4_. (b) Lattice parameters as a function of the Br content. (c) Lattice parameters as a function of Br distributed across the Wyckoff 4*a* site. (d) Percentage of the Br occupancy on the Wyckoff 4*d* site as a function of the total Br content. The data for Li_6_PS_5_Br in panels (b–d) are taken from ref (39). The arrow indicates the increase in Br content within Li_6–*x*_PS_5–*x*_Br_1+*x*_.

Rietveld refinement of the neutron diffraction patterns was used to determine the structure, especially with the aim to reveal the lithium substructure and the Br site disorder. The lattice parameter, occupancies (Br and S, Wyckoff 4*d* and 4*a*), sites (S, Wyckoff 16*e*), and thermal parameters (S, P, and Br) were refined. Figure 2a shows Rietveld refinement of slow-cooled Li_6_PS_5_Br; all structural tables can be found in Tables S1–S7. Samples (see Figure S1) contained less impurity phases of Li_3_PO_4_ and LiBr (less than 1.0 wt %), but these were assumed to have negligible impact on the ionic transport or structural analysis. While increasing the Br content up to *x* = 0.5, the cubic polymorphs were maintained. Hu et al.^21^ showed that increasing the Br content in Li_6–*x*_PS_5–*x*_Br_1+*x*_ (*x* = 0.7 and 0.8) led to the increase in LiBr and Li_3_PS_4_ impurities, indicating a solubility limit. Therefore, the analysis focused on the cubic polymorphs within the solubility limit (*x* ≤ 0.5).

The Pawley-fit-refined lattice parameter of slow-cooled Li_6_PS_5_Br results in *a* = 9.98356(7) Å, consistent with previous studies.^39^Figure 2b shows the lattice parameter as a function of the Br content. As the Br content increases (higher *x*), the lattice parameter decreases for the slow cooling method and increases for the fast cooling method. The difference in the systematic change in the lattice parameter as a function of the Br content and two cooling methods is noteworthy and unexpected. Figure 2c shows the lattice parameter as a function of Br distributed across the Wyckoff 4*a* site. Variation in the Br occupancy on the 4*d* site in Li_6_PS_5_Br can affect the lattice parameter due to the difference in ionic radii between bromide (196 pm) and sulfur (184 pm)^52^ anions occupying the 4*a* sites and changes in the Li^+^ occupancy around the 4*d*/4*a* sites as divalent sulfur is replaced by monovalent bromine.^39^ For the slow cooling method, increasing the Br content does not affect the Br occupancy at the 4*a* site significantly (Figure S3). Hence, the decrease in lattice parameter with increasing Br content may be due to the increase in Li^+^ vacancies or differences in Li^+^ distribution. In the quenched sample, a clear trend was shown between the lattice parameter and the Br occupancy in the 4*a* site. As the Br content increases, the Br occupancy in the 4*a* site also increases (Figure S3), resulting in a larger lattice parameter. This trend is consistent with the previous studies.^39^

The Br occupancy on the 4*d* site can be tuned in a single composition by adjusting the synthesis method.^39,53^ In this work, we employed a similar approach while increasing the Br content in lithium argyrodites. As the Br content (*x*) is increased, Br is distributed across the Wyckoff 4*a* and 4*d* sites in the crystal structure. Figure 2d shows the percentage of Br in the 4*d* site obtained by the two different cooling methods as a function of the total Br content. Slow-cooled samples have a lower Br 4*d* site occupancy, as determined by Rietveld refinements against neutron diffraction patterns. As the total Br content increases, Br in the 4*d* site increases, starting from 12% at *x* = 0.0 and reaching 63% at *x* = 0.5 for the slow-cooled method. The quenched method has an even higher Br in the 4*d* site, starting from 39% at *x* = 0.0–72% at *x* = 0.5, most likely because fast cooling “freezes in” the higher Br occupancy of the 4*d* site that is achieved at higher temperatures.

### Lithium Substructure

As mentioned above, in the lithium argyrodite structure, the anion framework creates 6 different types of tetrahedral interstices, five of which can potentially accommodate lithium.^29,31−33^ These tetrahedra are classified based on the number of shared corners, edges, or faces with adjacent PS_4_^3–^ units, and are referred to as types 1 through 5 (T1–T5).^37^ Li^+^ occupies only the type 5 and type 2 (T5, T2, and T5a) positions in Li_6_PS_5_Hal (Hal = Cl, Br, and I),^37,38,43^ forming cage-like structures around the central anion (Wyckoff 4*d*), as shown in Figure 1b. However, recent reports have indicated that Li^+^ can partially occupy the T4 site (Wyckoff 16*e*, Figure 1b) in Li_6.15_Al_0.15_Si_1.35_S_5.4_O_0.6_ and related Li_2_S-SnS_2_–SiS_2_–P_2_S_5_ sulfides.^42^ Additionally, other argyrodites showed the Li^+^ occupancy of T2 and T4 sites in “Li-deficient” materials with stoichiometries of <6.0 Li per formula unit.^40^ In order to study the lithium substructure in the Li_6–*x*_PS_5–*x*_Br_1+*x*_ series, we conducted a detailed investigation using neutron powder diffraction, analyzed by Rietveld refinement in the cubic *F*4̅3*m* space group. Figure S2 shows the occupancy of various Li sites in the argyrodite series by both quenching and slow cooling methods as a function of the bromide content. Rietveld refinement revealed 4 Li^+^ sites that are occupied depending on the composition of Li_6–*x*_PS_5–*x*_Br_1+*x*_: T5, T2, T5a, and T4. The overall Li^+^ content was constrained to obtain a charge balance with the refined Br content. For compositions *x* = 0.0–0.5 (quenching and slow cooling), the refined thermal displacement parameters of the T2 sites were relatively large compared to other Li^+^ sites in the structure (*B*_iso_ values of ∼9 Å^2^ for the T2 site), indicating a delocalization of Li^+^ around the T2 site. This high value is consistent with literature.^11,37,39,54^ The refined thermal displacement parameters of the T4 site were unphysically negative values for the slow-cooled *x* = 0.0 compositions. Additionally, a Fourier difference map showed no visible evidence of Li occupation on the T4 site. When the Br content increases in slow-cooled compositions up to *x* ≥ 0.3, additional T4 lithium sites were found to be occupied, as shown in Figure S2. These sites lead to a stable and improved fit (for example, when T4 sites are excluded in *x* = 0.3, this leads to an increase in *R*_wp_ from 6.4 to 7.0%).

**Figure 3:**
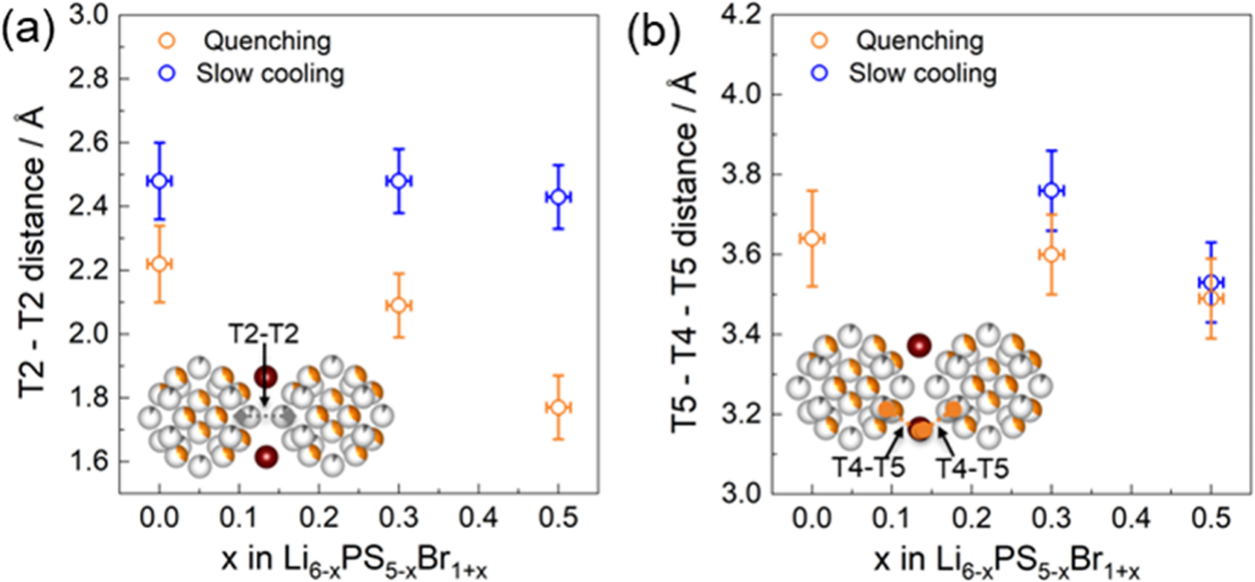
(a) T2–T2 distance and (b) T5–T4–T5 distance of Li_6–*x*_PS_5–*x*_Br_1+*x*_ as functions of the Br content. The inset shows a visual representation of lithium cages in which the T5–T4–T5 (orange dotted line) and T2–T2 (gray dotted line) jump distances offer a shorter distance between the lithium cages. The T2–T2 distance and 2 times of the T5–T4 distance decrease with increasing Br content, indicating greater connectivity between cages and increasing ionic conductivity.

The T4 sites have previously been identified in Cu and Ag argyrodites.^55,56^ Masuda et al.^40^ have reported on their occupation in mixed-halide argyrodite systems. The occupation of the T4 site, located between two cages, has been suggested to improve Li-ion diffusion in argyrodites. There are two intercage pathways which are important for long-range Li^+^ diffusion, as suggested in the literature:^35^ one pathway through two neighboring T2 sites (T2–T2) and another pathway between two T5 sites via the T4 site (T5–T4–T5), as shown in Figure 1c. Figure S4 shows that the T2, T5, and T4 polyhedral volumes are barely affected by a change in the Br content. The BVSE landscapes of the Li_6–x_PS_5–x_Br_1+x_ series reveal that cage-to-cage Li^+^ diffusion pathways are more connected for higher Br content (Figures S8 and S9). Figure 3 shows the T2–T2 and T5–T4–T5 distances as a function of bromide content using both slow cooling and quenching methods. The slow-cooled Li_6_PS_5_Br composition allows only for lithium-ion diffusion through T2–T2 pathways, where no occupancy of the T4 site is observed. As the bromine content increases, the T2–T2 distance remains constant for the slow-cooled method; however, it decreases for the quenching method. The T5–T4–T5 distances decrease with increasing bromide content in both synthesis methods, as depicted in Figure 3b. With increasing bromide content in the slow-cooled method, the T5–T4–T5 distance decreases from 3.76 (10) Å at *x* = 0.3 (composition) to 3.6 (9) Å at *x* = 0.5. In the quenched method, the T5–T4–T5 distance decreases from 3.64 (12) Å at *x* = 0.0 to 3.49 (8) Å at *x* = 0.5, as seen in Figure 3b. Summarizing, upon increasing the bromide content, the T5–T4–T5 intercage distance decreases for both slow and quenched cooled materials, and the T2–T2 intercage distance reduces only for the quenched cooling. This can potentially promote long-range diffusion, especially for the quenched cooled materials.

**Figure 4:**
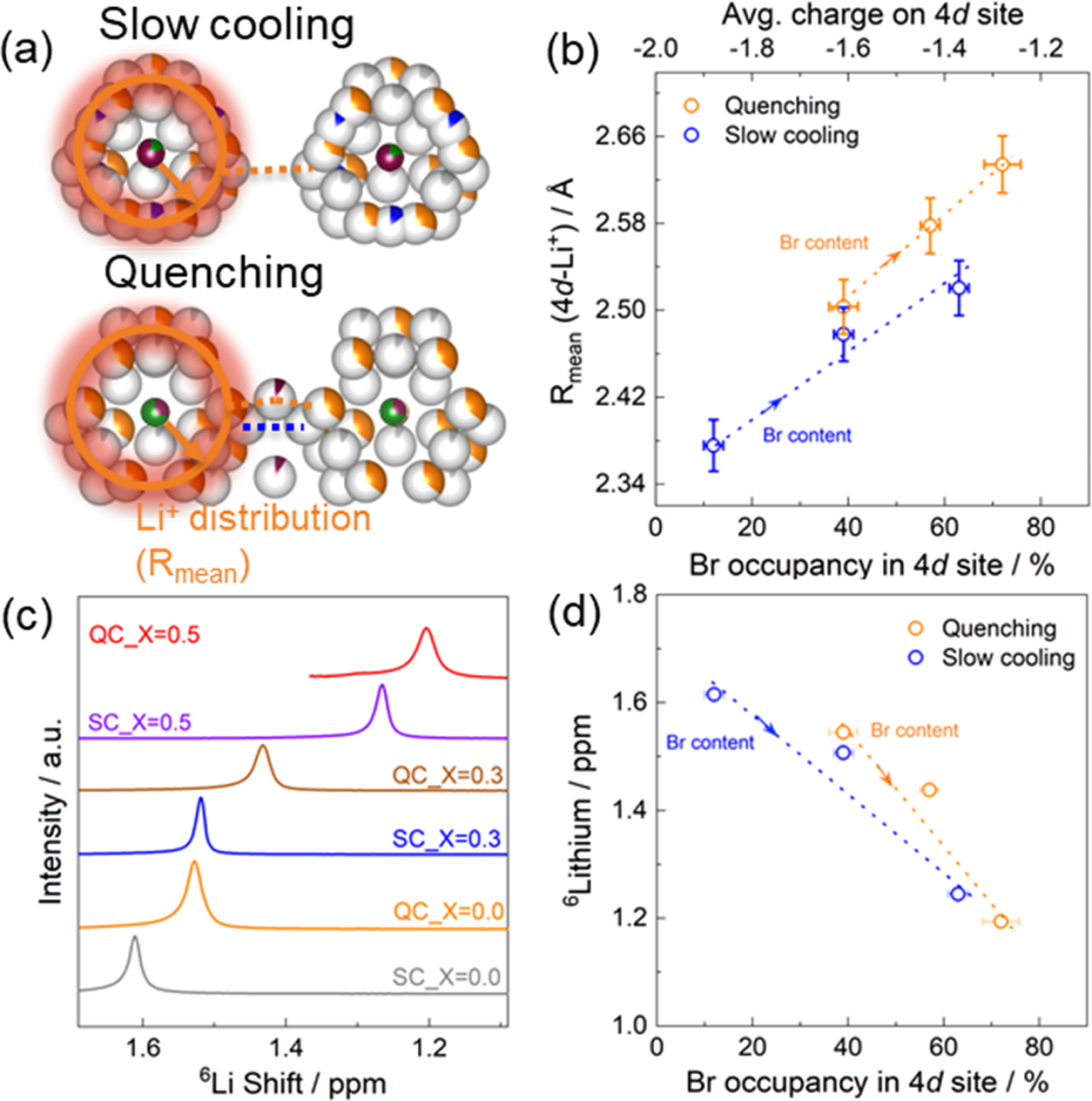
(a) Visual representation of *R*_mean_. (b) Average Li^+^ distribution, *R*_mean_, representing the radius of the sphere formed by Li positions centered around the anion position (Wyckoff 4*d*, S^2–^). As the Br content increases or the Br occupancy on the 4*d* site increases, the average charge on the 4*d* site decreases as the divalent S is replaced by the monovalent Br. This causes *R*_mean_ to increase, representing an expansion of the distance between Li^+^ cages. (c) ^6^Li NMR spectra and (d) ^6^Li shift ppm value of Li_6–*x*_PS_5–*x*_Br_1+*x*_ as a function of the Br content; the shift ppm values decrease with increasing Br content, further indicating a change in the local Li^+^ environment around the center of the anion (Wyckoff 4*d*, free sulfide anion site). The arrow indicates the increase in Br content within Li_6–*x*_PS_5–*x*_Br_1+*x*_.

The average charge on the Wyckoff 4*a* and 4*d* sites will vary based on their occupancy due to the different charges of Br^–^ and S^2–^. This can be anticipated to change the average surrounding Li^+^ substructure (occupancies). To quantify how Br^–^ impacts the arrangement of Li^+^, the average lithium distribution (denoted as *R*_mean_) in the structure was determined by calculating the average distance between the 4*d* site (central anion, S^2–^) and Li^+^ located at different sites (T2, T5, and T5a), weighed by the occupancy of each site within a single cage, as previously introduced.^37,39^ Previously, only the T5, T2, and T5a sites were considered,^39^ but at present the additional T4 site is included in the *R*_mean_ calculations. The visual representation of *R*_mean_ can be seen in Figure 4a. For the different total Br content and 4*d* Br^–^ occupancy, significant changes of *R*_mean_ are observed (Figure 4b). This result is supported by the room-temperature ^6^Li NMR spectra of Li_6–*x*_PS_5–*x*_Br_1+*x*_ (Figure 4c). The spectrum for slow-cooled Li_6_PS_5_Br displays an intense resonance at 1.62 ppm assigned to the Li^+^ atom occupying the T2, T5, and T5a sites. We observed that with increasing Br content, the ^6^Li resonance shifts toward lower ppm values (Figure 4d), as previously observed for a similar system.^21^ This shift to lower parts per million values indicates that the Li atoms are experiencing a more electron-rich environment. As the amount of Br^–^ on the 4*d* site increases, either through increasing the Br content, the average negative charge on the 4*d* site decreases as the divalent sulfide is replaced by the monovalent Br. This results in reduced electrostatic interactions between the electronegative 4*d* site and the electropositive Li atoms. The Li atoms move away, causing *R*_mean_ to increase. More charge is maintained on the Li atom as its electrons experience less interaction with the 4*d* site, causing the ^6^Li shift to move to lower ppm values.^61^ The increased Li cage radius is the origin of the reduced T2–T2 and T5–T4–T5 distances, effectively the result of the Br distribution over the 4*a* and 4*d* sites, which can be expected to result in better cage connectivity and thus higher overall conductivity.^39,40^

### Ionic Transport

**Figure 5:**
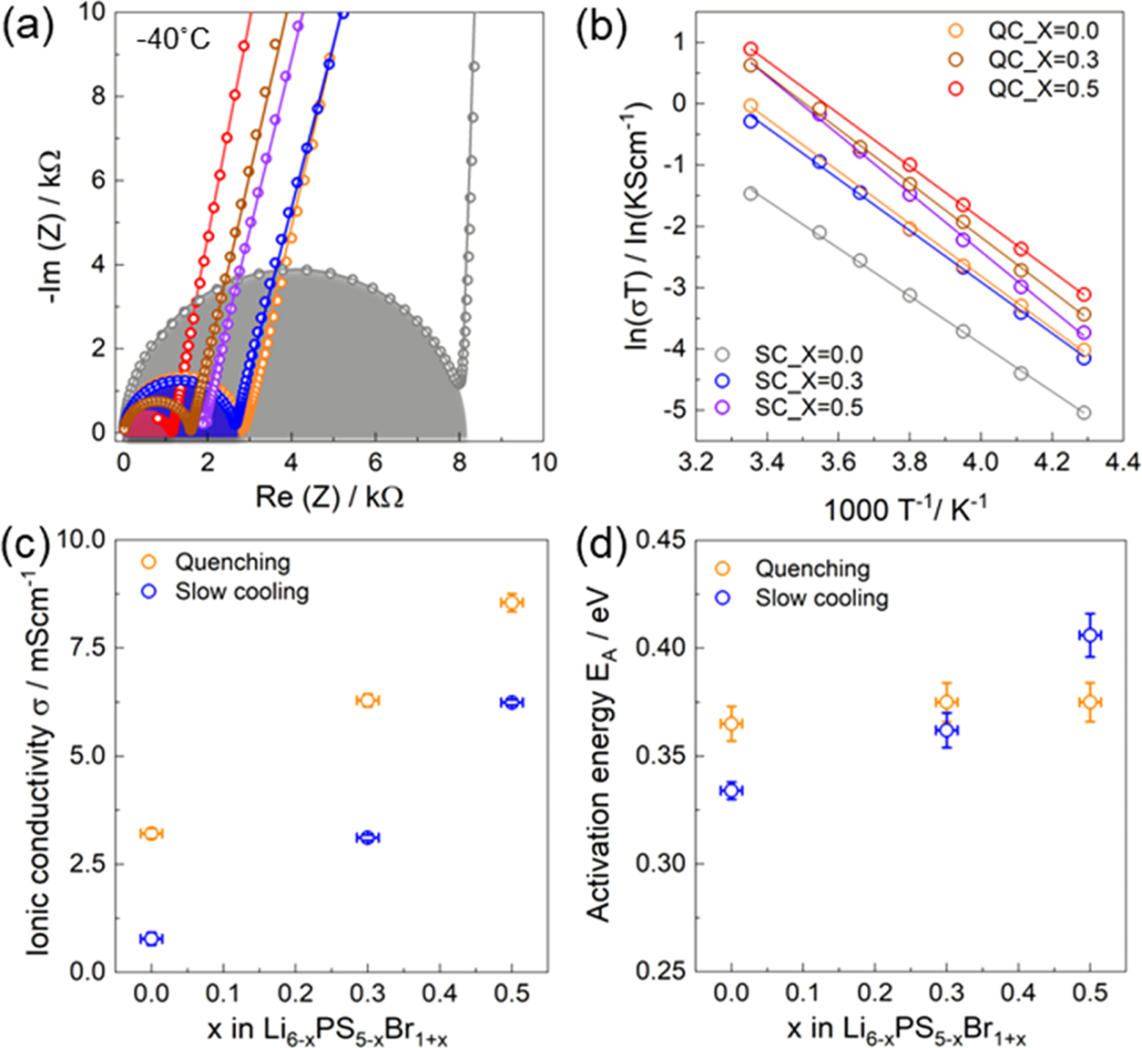
(a) Impedance response of the Li_6–*x*_PS_5–*x*_Br_1+*x*_ series at a temperature of −40 °C. The open circles indicate the impedance response and exhibited capacitances of around 1–8 × 10^–10^ F/cm^2^ and an α value of 0.89–0.97, corresponding to bulk transport. (b) Arrhenius plots of the Li_6–*x*_PS_5–*x*_Br_1+*x*_ series, obtained from temperature-dependent impedance spectroscopy. (c) Ionic conductivities at room temperature and (d) activation energy *E*_A_ of Li_6–*x*_PS_5–*x*_Br_1+*x*_ as a function of the Br content.

De Klerk et al.^35^ suggested that a higher site disorder up to 75% or a higher halide content in argyrodites can enhance ionic conductivity by facilitating intercage diffusion and by providing a more optimal Li-vacancy distribution.^24−26,35,43,57−59^ To examine the effect of varying the total Br content and Br 4*d* occupancy on the ionic conductivity of Li_6–*x*_PS_5–*x*_Br_1+*x*_, we performed temperature-dependent impedance spectroscopy to measure the ionic conductivity and activation energy. Figure 5a shows the Nyquist plots of quenched and slow-cooled Li_6*–x*_PS_5–*x*_Br_1+*x*_ at −40 °C, and other temperatures can be found in Figure S5. The impedance data at lower temperatures (−40 to 0 °C) were fitted with a resistor and constant phase element (CPE) in parallel; another CPE in the series represents the blocking electrode. The resistor represents the overall ionic conductivity. At higher temperatures (10 and 25 °C), the response for the CPE/resistor shifts to higher frequencies and is difficult to resolve within the measured frequency range. In this case, only the tails of the blocking electrodes were used for the fit. The CPE in parallel exhibited a capacitance of around 1 to 8 × 10^–10^ F/cm^2^, indicating bulk transport.^39^ The impedance *Z* of CPE is given by  where ω is the angular frequency, *J* is the imaginary unit, *Q* is a parameter related to the magnitude of the impedance, and α represents the phase constant. The α value of 0.89–0.97 represents the ideality of the semicircle, with an α value of 1 indicating an ideal capacitor. Figure 5c shows the ionic conductivities at room temperature as a function of the Br content. The ionic conductivity increases with the Br content for both cooling methods. As previously shown, a higher site disorder (4*d* Br occupancy) leads to an improvement of the ionic conductivity.^39^ In this work, the conductivity of the slow-cooled Li_6_PS_5_Br sample (12% Br occupancy on the 4*d* site) is 0.78 mS/cm. The conductivity of the slow-cooled samples increases from 0.78 to 6.2 mS/cm with increasing Br content, while the conductivity of quenched samples increases from 3.21 to 8.55 mS/cm with increasing Br content. Figure 5b shows that the temperature dependence of the ionic conductivities exhibits a linear Arrhenius behavior from which an activation energy can be obtained. As shown in Figure 5d, the activation energy increases slightly with increasing Br content in both the slow-cooled and quenched samples. The observed increase in the activation barrier, concurrent with an increase in conductivity, may seem paradoxical at first. However, this behavior can be explained by the enthalpy–entropy compensation rule, also referred to as the Meyer–Neldel rule.^60−63^ This rule states that an increase in the Arrhenius prefactor can lead to an increase in the activation barrier, as demonstrated in Figures S6 and S7, effectively meaning that more jumps occur, presumably because of the more optimal Li^+^ distribution over the sites, providing a higher conductivity.

### Structure–Property Relationship

**Figure 6:**
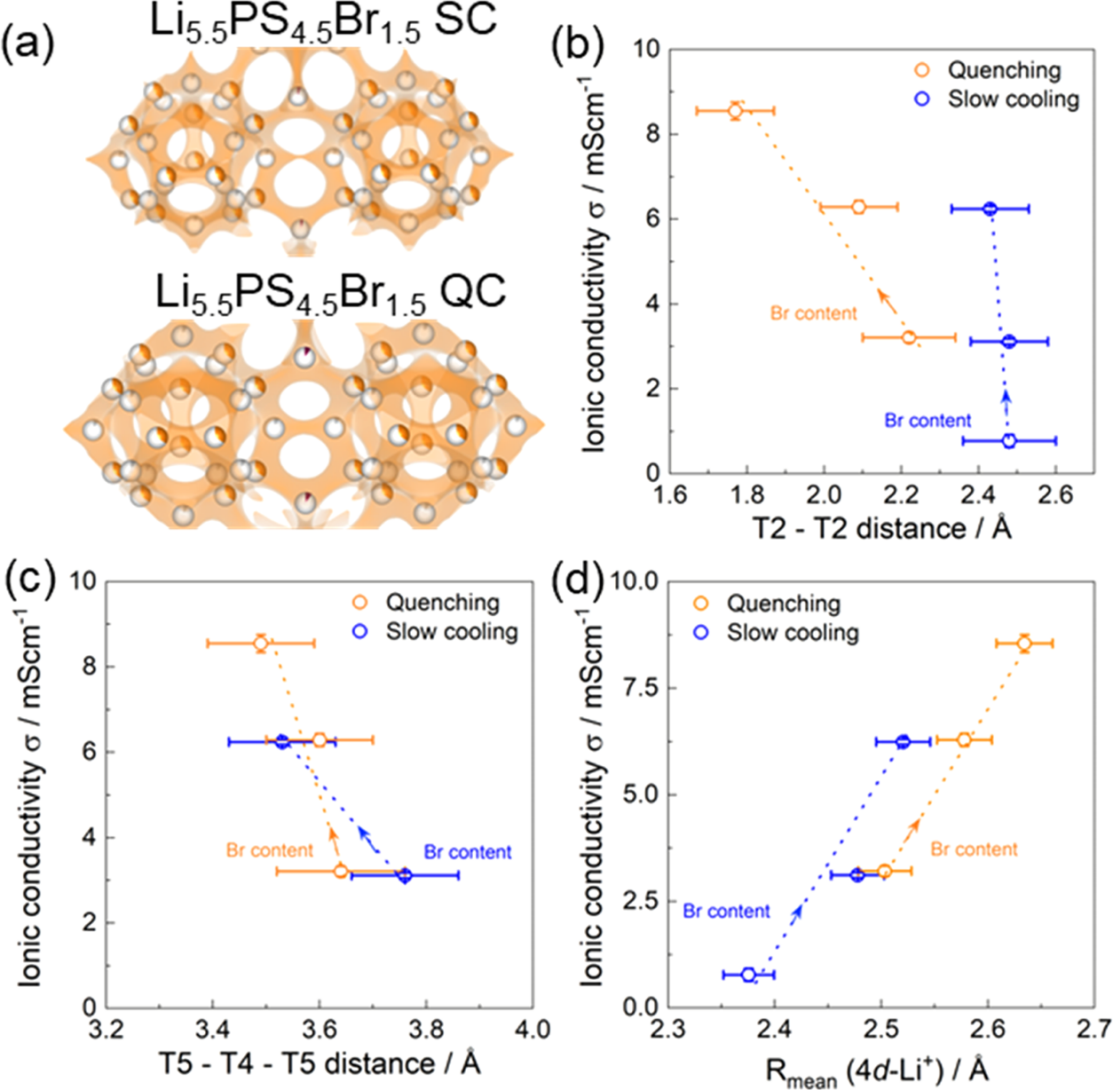
(a) Lithium diffusion pathways analyzed using the BVSE of slow-cooled Li_5.5_PS_4.5_Br_1.5_ and quenched Li_5.5_PS_4.5_Br_1.5_ compositions. The room-temperature ionic conductivity is shown as a function of (b) the T2–T2 distance, (c) the T5–T4–T5 distance, and (d) the *R*_mean_ distance. As the *R*_mean_ value increases, it indicates an expansion of the Li cage away from its center, which supports more connections between the cages through the T2–T2 and T5–T4–T5 distances. BVSE analyses show that cage-to-cage Li^+^ diffusion pathways are more connected with a higher Br content. For the slow-cooled method, the T2–T2 distance remains constant, the T5–T4–T5 distance decreases, and the T4 Li occupancy increases, leading to more connections between cages and an increase in ionic conductivity from 0.78 to 6.20 mS/cm. In the quenched method, both the T5–T4–T5 and T2–T2 distances decrease with increasing Br content. Additionally, the T4 Li occupancy increases, resulting in an increase in connections between lithium cages and an increase in ionic conductivity from 3.21 to 8.55 mS/cm. The arrow indicates the increase in Br content within Li_6–*x*_PS_5–*x*_Br_1+*x*_.

Increasing the 4*d* Br occupancies through the synthesis method can significantly improve the ionic conductivity in lithium argyrodites. The occupancy and diffusion pathway and kinetics of lithium, within the Li cage and between the Li cages, are dictated by the halogen content and distribution over the 4*d* and 4*a* sites. In this study, we were able to tune the Br occupancies in the 4*d* site and the lithium substructure in each composition, leading to the following observations:

- The slow cooling method leads to a decrease in the lattice parameter, while the fast cooling method leads to an increase in the lattice parameter as the bromide content increases. The slow cooling method does not affect the Br occupancy at the 4*a* site, but decreasing the Li content and differences in Li^+^ distribution leads to a decrease in the lattice parameter. As the Br content increases in the quenched sample, the Br occupancy at the 4*a* site increases, which leads to a larger lattice parameter.
- As the total Br content increases, the 4*d* Br occupancies also increase, starting from 12 to 63% for the slow-cooled method. The 4*d* Br occupancy is larger at higher temperatures and quenching “freezes in” the higher 4*d* Br occupancies at room temperature.^53^ The quenching method therefore results in even higher 4*d* Br occupancies, ranging from 39 to 72%. Overall, this work highlights the importance of the synthesis methods as a tool to control the 4*d* Br occupancies in lithium argyrodites, which plays a key factor in the Li-ion conductivity.
- In the lithium substructure, the observation of nonzero T4 occupation implies the activation of a new pathway for Li-ion diffusion, consisting of shorter intercage Li^+^ jumps through face-sharing tetrahedral environments. This alternative pathway is in addition to the T2–T2 jumps that are observed in most lithium argyrodites. The intercage distance, which is connecting neighboring lithium cages via the T5–T4–T5 or T2–T2 distance, appears crucial to increase the ionic conductivity. Consistently, the BVSE analyses show that cage-to-cage Li^+^ diffusion pathways are more connected for a higher Br content (Figures 6a, S8, and S9).
- While the 4*d* Br occupancy increases for both cooling methods (see above section), the average negative charge on the 4*d* site decreases as the divalent sulfide is replaced by the monovalent bromide ion. This reduces the electrostatic interaction between the electronegative 4*d* site and the electropositive Li ions. The Li ions move away, causing *R*_mean_ to increase and subsequently, more charge is maintained on the Li ion, causing the observed ^6^Li shift to move to lower ppm values. The increased Li cage radius (Figure 6d) is the origin of the reduced T2–T2 and T5–T4–T5 distances, and effectively the result of the difference in Br distribution over the 4*d* sites, which is held responsible for better connectivity between the cages, resulting in the larger overall conductivity observed.
- As the bromide content increases for the slow-cooled samples, the T2–T2 distance remains constant. The T5–T4–T5 distances decrease as the T4 Li occupancy increases (Figure 6b,c), resulting in higher connectivity between cages and increasing the ionic conductivity from 0.78 to 6.20 mS/cm.
- For quenched samples, the T5–T4–T5 and T2–T2 distances both decrease with increasing Br content. Additionally, the T4 Li occupancy increases, leading to an increase in the diffusion pathways between the lithium cages and in a reduced intercage jump distance (T2–T2 and T5–T4–T5), resulting in an increase in ionic conductivity from 3.21 to 8.55 mS/cm. Hereby, we highlight how the bromide content and sublattice occupancy affect the Li cage expansion and the Li occupancies, which can be used to improve the diffusion pathway and Li-ion mobility and thus the Li-ion conductivity. We anticipate that understanding these structure–conductivity relationships further enhances the application potential of lithium argyrodites in solid-state lithium-ion batteries.

## Conclusions

In this study, we synthesized bromide-enriched lithium argyrodites (Li_6–*x*_PS_5–*x*_Br_1+*x*_) with compositions ranging from *x* = 0.0 to 0.5 using two cooling methods. It is found that the cooling method controls the Br occupancy on the 4*d* site in the Li_6–*x*_PS_5–*x*_Br_1+*x*_ series. Rietveld refinement of the neutron diffraction patterns reveals how this affects the 4*d* and 4*a* site occupancies and the lithium substructure. As the Br content increased, the Li^+^ ions moved away from the center of the cage (4*d* site), as captured by the *R*_mean_. Additionally, this induces T4 site occupancy, which opens up an alternative Li-ion diffusion pathway with a lower activation energy. The average negative charge on the 4*d* site decreases due to an increase in the 4d Br^–^ occupancy, resulting in an increased Li cage radius and reduced T2–T2 and T5–T4–T5 distances, leading to a better connected Li^+^ landscape. The slow-cooled method results in an increase in ionic conductivity from 0.78 mS/cm at *x* = 0.0–6.2 mS/cm at *x* = 0.5, while the quenched method shows an increase from 3.21 mS/cm at *x* = 0.0–8.55 mS/cm at *x* = 0.5. These findings demonstrate the importance of understanding how chemical changes and synthesis conditions affect the lithium substructure in order to tailor the transport properties of these materials and increase their potential for solid-state batteries.

## Supporting Information Available

All structural data as obtained from Rietveld refinements of neutron diffraction data; lithium occupancies; Br occupancies on the Wyckoff *4a* site and Wyckoff (*4a* + *4d*) sites; Li T5, Li T2, and Li T4 volumes; electrochemical impedance data; Arrhenius prefactors vs Br content; Meyer–Neldel plot of the Arrhenius prefactors and activation barriers; and BVSE of the slow-cooled and quenched Li_6–*x*_PS_5–*x*_Br_1+*x*_ series (PDF)
