# Supplementary material for: Exploring the Relationship Between Halide Substitution, Structural Disorder, and Lithium Distribution in Lithium Argyrodites (Li6–xPS5–xBr1+x)
Source: Chem Mater. 2023 Sep 18;35(19):8081–91. doi: 10.1021/acs.chemmater.3c01525 (PMC10569443; doi:10.1021/acs.chemmater.3c01525)
Supplement: Supplementary file 1 — cm3c01525_si_001.pdf [file cm3c01525_si_001.pdf]

Supporting Information -

Exploring the Relationship Between Halide Substitution, Structural Disorder, and Lithium Distribution in Lithium Argyrodites ($Li_{6-x}PS_{5-x}Br_{1+x}$)

Ajay Gautam*, Hanan Al-Kutubi, Theodosios Famprikis, Swapna Ganapathy, Marnix Wagemaker*

Storage of Electrochemical Energy, Department of Radiation Science and Technology, Faculty of Applied Sciences, Delft University of Technology, Mekelweg 15, 2629JB, Delft, The Netherlands

Email:m.wagemaker@tudelft.nl

Email:A.ajaygautam@tudelft.nl

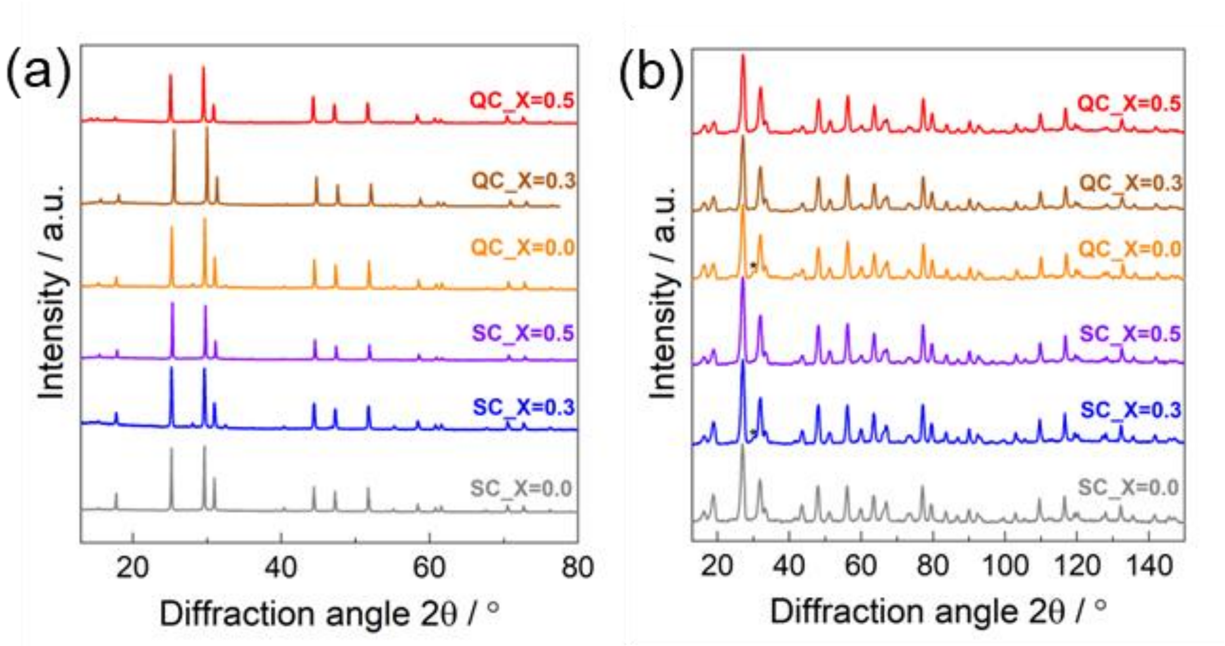

Figure S1:(a) X-ray diffraction and (b) neutron diffraction patterns of $Li_{6-x}PS_{5-x}Br_{1+x}$ as a function of Br content for the slow-cooled "SC" and quench cooled "QC" methods.

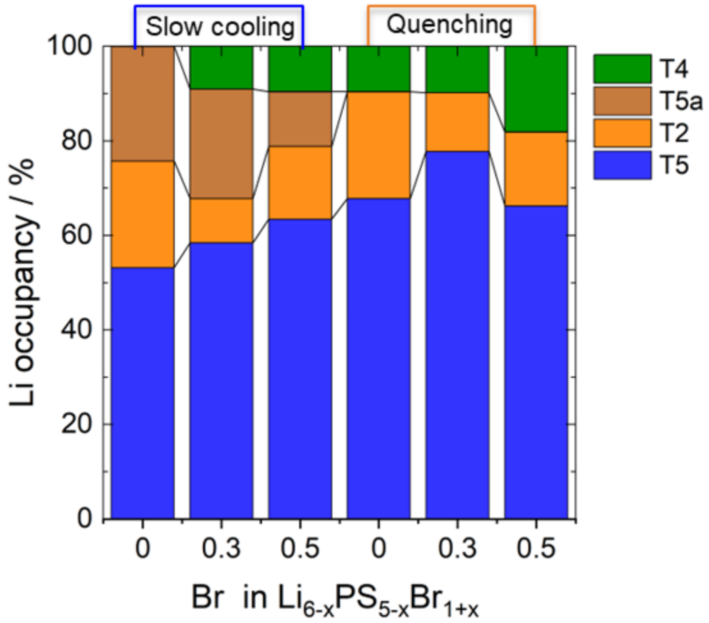

Figure S2: The Li^+ distribution across the different sites of $Li_{6-x}PS_{5-x}Br_{1+x}$ as a function of Br content.

Table S1. Constraints used to refine $Li_{7-x}PS_{6-x}Br_{1+x}$ neutron diffraction data.

Atom	Wyckoff site	*x/a*	y/*b*	z/*c*	Occ	B_{eq}/ Å^2
**LiT5**	48*h*	*Pos1*	*Pos2*	*1-Pos1*	*Occ1*	*Var1*
**LiT2**	48*h*	*Pos3*	*Pos4*	*0.5+Pos4*	*((7-(1-Occ3+Occ4))*4-48*Occ1-24*Occ5-16*Occ6)/48*	*Var2*
**LiT5a**	24*g*	0.25	Pos5	0.75	*Occ5*	*Var1*
**LiT4**	16*e*	*Pos6*	*Pos6*	*Pos6*	*Occ6*	*Var3*
**Br1**	4*a*	0	0	1	*Occ3*	*Var4*
**Br2**	4*d*	*0.25*	*0.25*	*0.75*	*1-Occ4*	*Var5*
**P1**	4*b*	0	0	0.5	1	*Var6*
**S1**	4*d*	0.25	0.25	0.75	*Occ4*	*Var5*
**S2**	16*e*	*Pos7*	*-Pos7*	*0.5+Pos7*	*1*	*Var7*
**S3**	4*a*	0	0	1	*1-Occ3*	*Var4*

Table S2. Crystallographic data of slow-cooled Li_6PS_5Br sample. The lattice parameter, atomic positions, and occupancies are obtained from the Rietveld refinements against the neutron diffraction data. The uncertainty/error value is shown in brackets.

**a = 9.9835(2) Å; 0.52 wt % Li_3PO_4** **R_{wp} = 6.0 % ; λ = 1.667 Å**						
**Atom**	Wyckoff site	*x/a*	y/*b*	z/*c*	Occ	B_{eq}/ Å^2
**LiT5**	48*h*	0.3036(1)	0.0231(2)	0.6964(1)	0.30(2)	2.2(3)
**LiT2**	48*h*	0.296(7)	0.087(6)	0.587(6)	0.13(2)	14(4)
**LiT5a**	24*g*	0.25	0.013(6)	0.75	0.135(6)	2.1(4)
**LiT4**	*16e*	--	--	--	--	--
**Br1**	4*a*	0	0	1	0.90(2)	2.92(2)
**Br2**	4*d*	0.25	0.25	0.75	0.12(2)	1.6(2)
**P1**	4*b*	0	0	0.5	1	2.92(2)
**S1**	4*d*	0.25	0.25	0.75	0.88(2)	1.44(4)
**S2**	16*e*	0.1185(4)	-0.1185(4)	0.6185(4)	1	2.02(7)
**S3**	4*a*	0	0	1.0	0.10(2)	1.6(2)

Table S3. Crystallographic data of slow-cooled $Li_{5.7}PS_{4.7}Br_{1.3}$ sample. The lattice parameter, atomic positions, and occupancies are obtained from the Rietveld refinements against the neutron diffraction data. The uncertainty/error value is shown in brackets.

***a* = 9.9826(2) Å; 0.4 wt % Li_3PO_4; 1.8 wt % LiBr**

R_{wp} = 6.4 % ; λ = 1.667 Å

**Atom**	Wyckoff site	*x/a*	y/*b*	z/*c*	Occ	B_{eq}/ Å^2
**LiT5**	48*h*	0.0306(9)	0.0207(1)	0.6937(1)	0.324(8)	3.5(2)
**LiT2**	48*h*	0.2927(9)	0.0862(7)	0.58	0.073(2)	10(2)
**LiT5a**	24*g*	0.25	0.011(7)	0.75	0.126(2)	2.9(3)
**LiT4**	16e	0.162(9)	0.162(9)	0.162(9)	0.044(7)	3.0(2)
**Br1**	4*a*	0	0	1	0.88(2)	2.68(9)
**Br2**	4*d*	0.25	0.25	0.75	0.40(1)	2.11(9)
**P1**	4*b*	0	0	0.5	1	1.46(5)
**S1**	4*d*	0.25	0.25	0.75	0.60(2)	2.11(9)
**S2**	16*e*	0.1181(3)	-0.1181(3)	0.6181(3)	1	2.38(6)
**S3**	4*a*	0	0	1.0	0.12(2)	2.68(9)

Table S4. Crystallographic data of slow-cooled $Li_{5.5}PS_{4.5}Br_{1.5}$ sample. The lattice parameter, atomic positions, and occupancies are obtained from the Rietveld refinements against the neutron diffraction data. The uncertainty/error value is shown in brackets.

***a* = 9.9742(2) Å; 0.6 wt % LiBr**

R_{wp} = 6.0 % ; λ = 1.667 Å

Atom	Wyckoff site	*x/a*	y/*b*	z/*c*	Occ	B_{eq}/ Å^2
**LiT5**	48*h*	0.309(1)	0.0147(2)	0.690(1)	0.326(9)	3.7(8)
**LiT2**	48*h*	0.283(3)	0.0855(7)	0.5854(7)	0.084(3)	14(3)
**LiT5a**	24*g*	0.25	0.015(2)	0.75	0.065(6)	3.8(4)
**LiT4**	16*e*	0.155(8)	0.155(8)	0.155(8)	0.05(1)	3.8(5)
**Br1**	4*a*	0	0	1	0.87(3)	2.32(9)
**Br2**	4*d*	0.25	0.25	0.75	0.64(3)	2.33(9)
**P1**	4*b*	0	0	0.5	1	2.11(1)
**S1**	4*d*	0.25	0.25	0.75	0.36(3)	2.33(9)
**S2**	16*e*	0.1186(3)	-0.1186(3)	0.6186(3)	1	3.2(1)
**S3**	4*a*	0	0	1.0	0.13(3)	2.32(9)

Table S5. Crystallographic data of quenched Li_6PS_5Br sample. The lattice parameter, atomic positions, and occupancies are obtained from the Rietveld refinements against the neutron diffraction data. The uncertainty/error value is shown in brackets.

***a* = 9.9592(2) Å; 0.81 wt % Li_3PO_4; 2.07 wt % LiBr**

R_{wp} = 3.59 % ; λ = 1.667 Å

Atom	Wyckoff site	*x/a*	y/*b*	z/*c*	Occ	B_{eq}/ Å^2
**LiT5**	48*h*	0.3088(9)	0.0181(1)	0.6912(9)	0.359	3.9(4)
**LiT2**	48*h*	0.300(7)	0.0789(5)	0.5789(5)	0.12(2)	13(4)
**LiT5a**	24*g*	--	--	--	--	--
**LiT4**	16e	0.158(7)	0.158(7)	0.158(7)	0.05(1)	3.89(4)
**Br1**	4*a*	0	0	1	0.64(2)	2.6(1)
**Br2**	4*d*	0.25	0.25	0.75	0.39(2)	2.1(1)
**P1**	4*b*	0	0	0.5	1	1.52(5)
**S1**	4*d*	0.25	0.25	0.75	0.61(2)	2.1(1)
**S2**	16*e*	0.1182(1)	-0.1182(1)	0.6182(1)	1	2.39(5)
**S3**	4*a*	0	0	1.0	0.36(2)	2.6(1)

Table S6. Crystallographic data of quenched $Li_{5.7}PS_{4.7}Br_{1.3}$ sample. The lattice parameter, atomic positions, and occupancies are obtained from the Rietveld refinements against the neutron diffraction data. The uncertainty/error value is shown in brackets.

a = 9.9718(2) Å; 0.27 wt % Li_3PO_4

R_{wp} = 5.10 % ; λ = 1.667 Å

Atom	Wyckoff site	*x/a*	y/*b*	z/*c*	Occ	B_{eq}/ Å^2
**LiT5**	48*h*	0.306(1)	0.178(2)	0.0694(1)	0.396(2)	4.9(5)
**LiT2**	48*h*	0.2699(9)	0.074(9)	0.574(7)	0.063(2)	9(3)
**LiT5a**	24*g*	--	--	--	--	--
**LiT4**	16e	0.154(2)	0.154(2)	0.154(2)	0.05(1)	4.8(4)
**Br1**	4*a*	0	0	1	0.72(2)	2.27(9)
**Br2**	4*d*	0.25	0.25	0.75	0.58(2)	2.13(9)
**P1**	4*b*	0	0	0.5	1	1.75(4)
**S1**	4*d*	0.25	0.25	0.75	0.42(2)	2.13(9)
**S2**	16*e*	0.1187(2)	-0.1187(2)	0.6940(8)	1	2.31(4)
**S3**	4*a*	0	0	1.0	0.28(2)	2.27(9)

Table S7. Crystallographic data of quenched $Li_{5.5}PS_{4.5}Br_{1.5}$ sample. The lattice parameter, atomic positions, and occupancies are obtained from the Rietveld refinements against the neutron diffraction data. The uncertainty/error value is shown in brackets.

***a* = 9.9777(2) Å; 0.79 wt % Li_3PO_4**

R_{wp} = 5.87 %; λ = 1.667 Å

**Atom**	Wyckoff site	*x/a*	y/*b*	z/*c*	Occ	B_{eq}/ $Å^2$
**LiT5**	48*h*	0.3099(9)	0.0151(1)	0.6901(9)	0.34(1)	3.96(5)
**LiT2**	48*h*	0.288(3)	0.074(2)	0.574(2)	0.084(2)	13(2)
**LiT5a**	24*g*	--	--	--	--	--
**LiT4**	16*e*	0.152(4)	0.152(4)	0.152(4)	0.093(2)	3.90(5)
**Br1**	4*a*	0	0	1	0.78(2)	1.94(1)
**Br2**	4*d*	0.25	0.25	0.75	0.72(3)	2.35(1)
**P1**	4*b*	0	0	0.5	1.0	2.35(1)
**S1**	4*d*	0.25	0.25	0.75	0.28(3)	2.35(1)
**S2**	16*e*	0.11774(1)	-0.11774(1)	0.61774(1)	1.0	3.2(1)
**S3**	4*a*	0	0	1.0	0.22(2)	1.94(1)

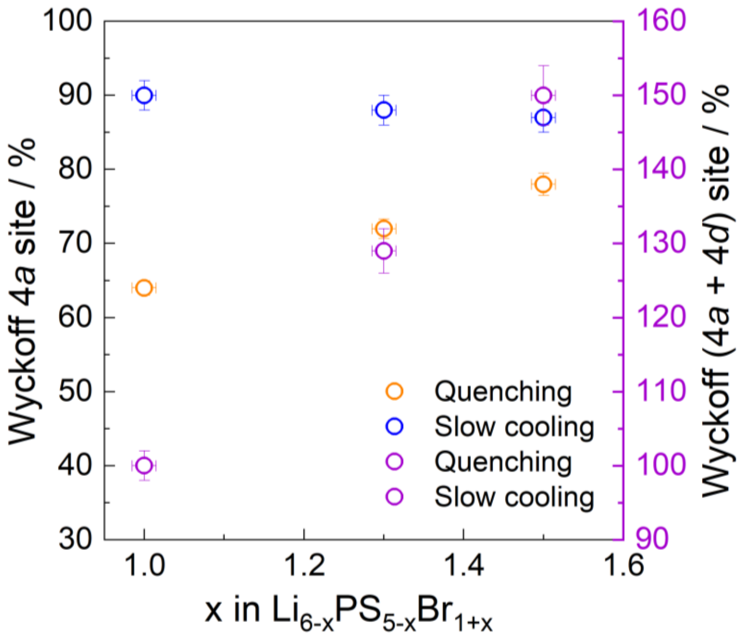

Figure S3: Percentage of Br content distributed across Wyckoff 4a site, and total Br content in each composition ($Li_{6-x}PS_{5-x}Br_{1+x}$).

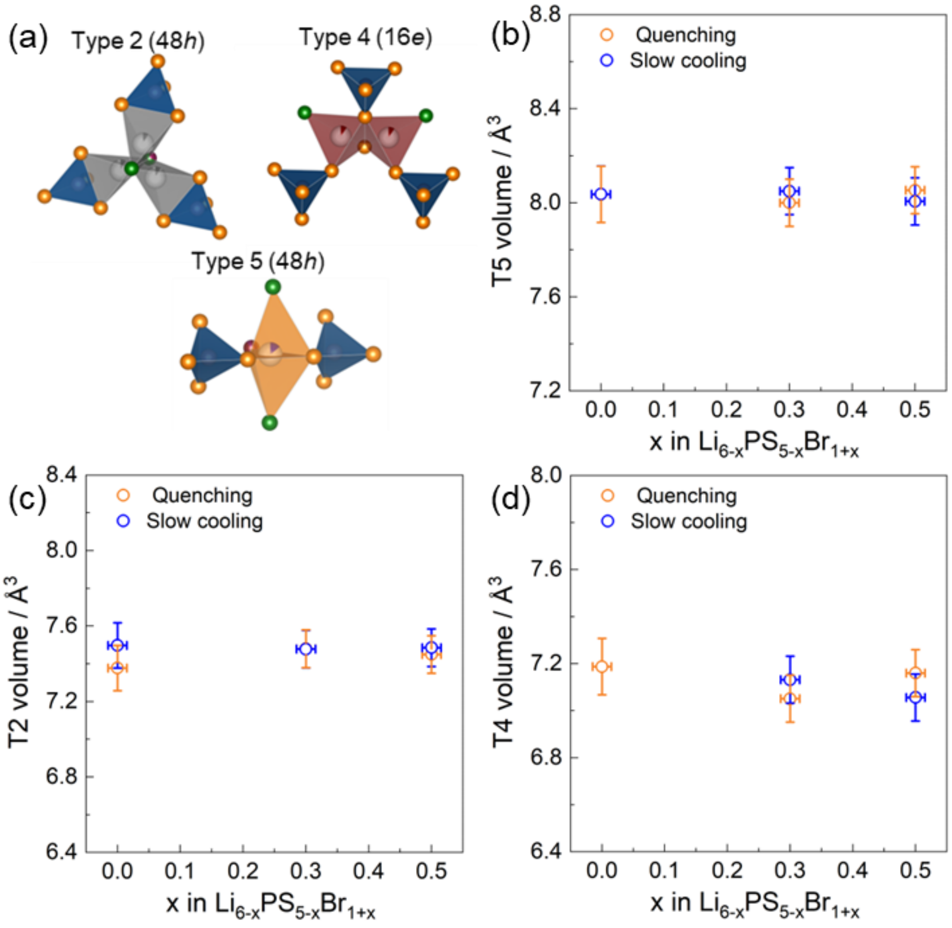

Figure S4: (a) Lithium diffusion pathway showing the tetrahedral face-connection between the T2 and T4 site that connects the different Li^+ *cages (proposed in ref .*[1] *). (b) T5, (c) T2, and (d) T4 volume as a function of Br content.*

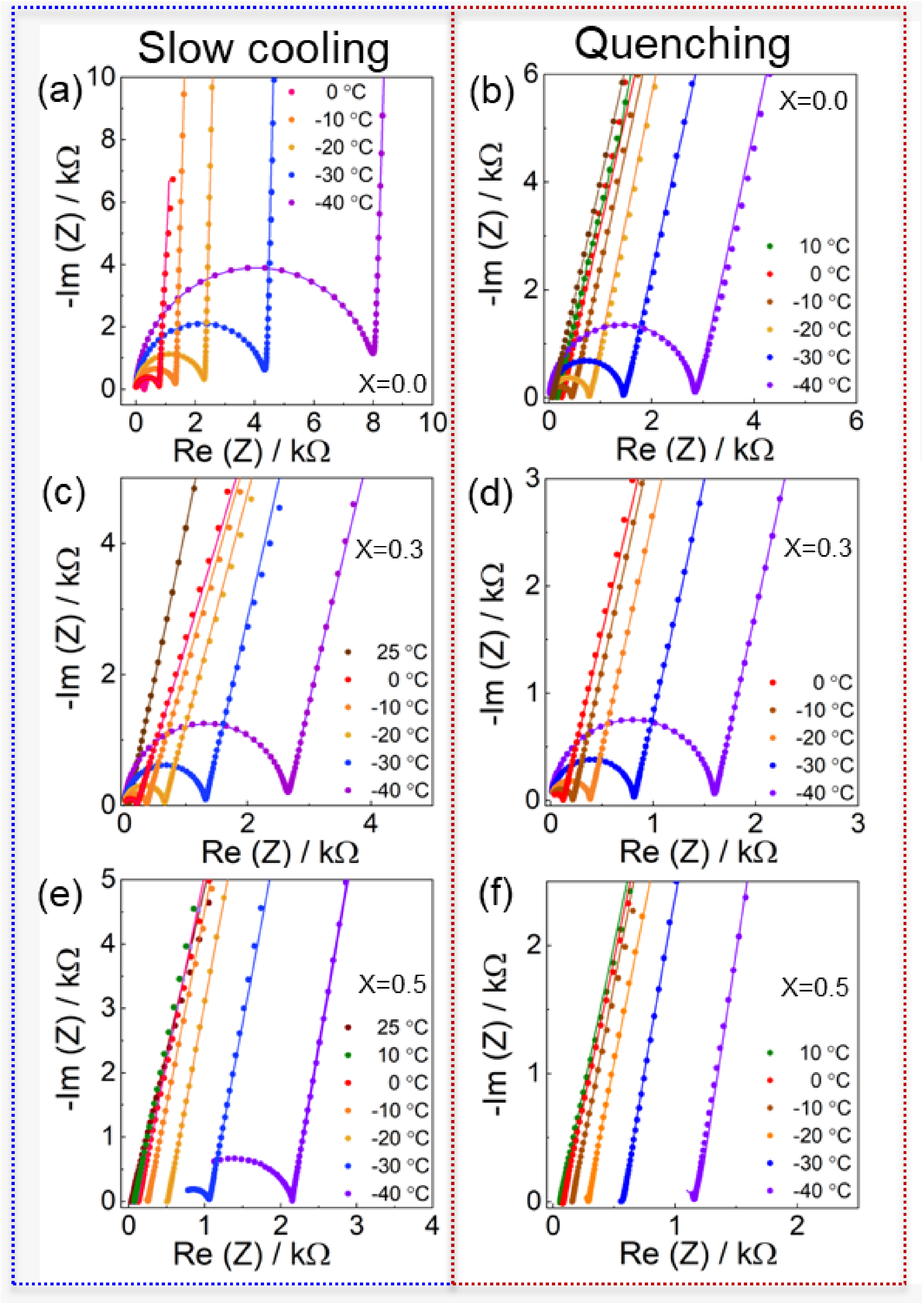

Figure S5: (a) Nyquist plots of the impedance response of the $Li_{6-x}PS_{5-x}Br_{1+x}$ *series at a temperature range of -40°C to 10°C. The closed circles indicate the impedance data and line representing the obtained impedance, exhibited capacitances of around 1 to 8 x* 10^{-10} *F* cm^{-2}*, and an α value of 0.89−0.9, corresponding to bulk transport.*

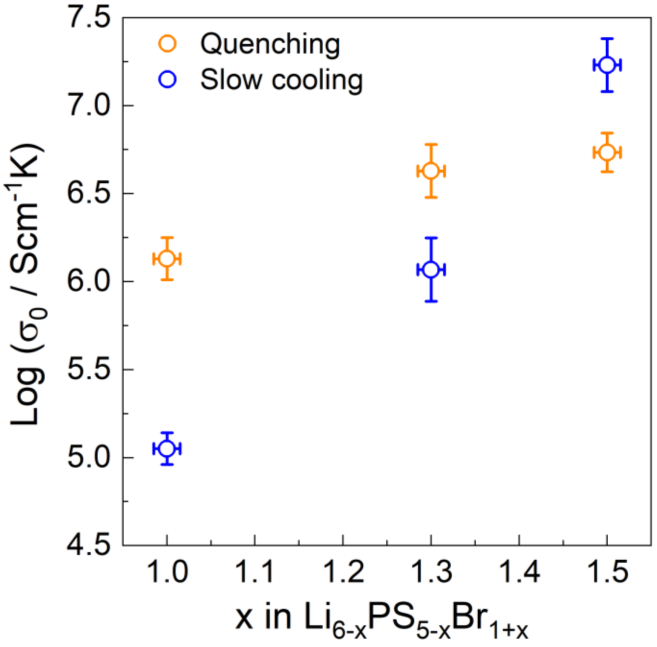

Figure S6: Pre-factor (σ_0) obtained from Arrhenius fits of the conductivity obtained from EIS using the equation (σ) = σ_0 exp(-E_A/kT)), for varying levels of Br content, slow cooling, and quenched compositions.

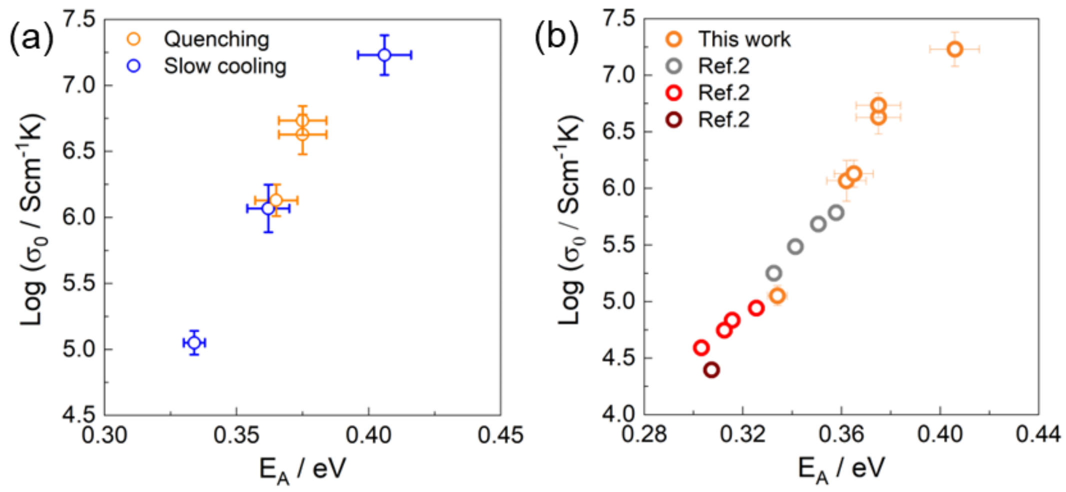

Figure S7: Meyer-Neldel plot of log (σ_0) vs. E_A, showing the enthalpy-entropy compensation of the pre-factor with the activation barrier. The data for Li_6PS_5Br are taken from ref.[2].

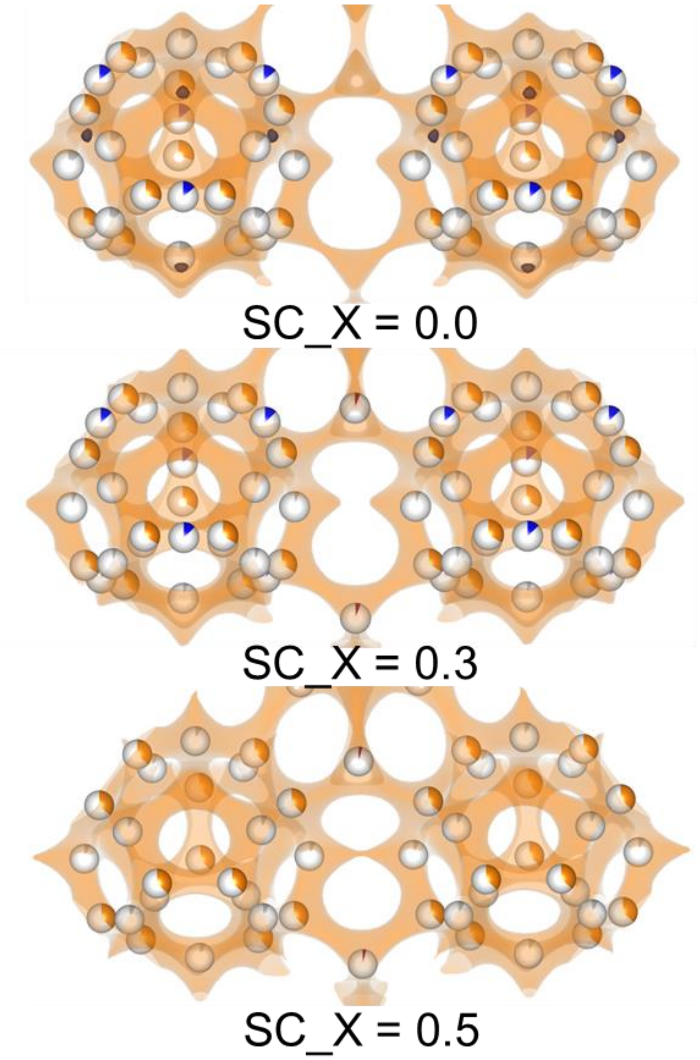

Figure S8: BVSE lithium diffusion pathway of slow cooling compositions x=0.0, 0.3, and 0.5($Li_{6-x}PS_{5-x}Br_{1+x}$)

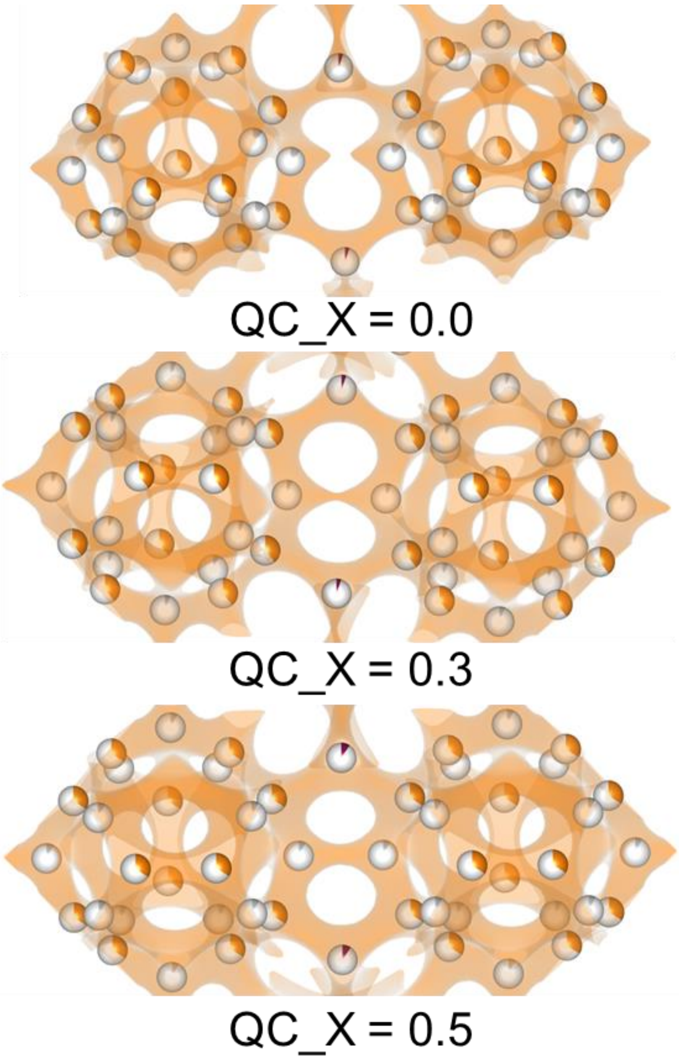

Figure S9: BVSE lithium diffusion pathway of quenching compositions x=0.0, 0.3, and 0.5 ($Li_{6-x}PS_{5-x}Br_{1+x}$).

Reference;

(1) Kong, S. T.; Deiseroth, H. J.; Reiner, C.; Gün, Ö.; Neumann, E.; Ritter, C.; Zahn, D. Lithium Argyrodites with Phosphorus and Arsenic: Order and Disorder of Lithium Atoms, Crystal Chemistry, and Phase Transitions. *Chemistry - A European Journal* **2010**, *16* (7), 2198–2206. https://doi.org/10.1002/chem.200902470.

(2) Gautam, A.; Sadowski, M.; Prinz, N.; Eickhoff, H.; Minafra, N.; Ghidiu, M.; Culver, S. P.; Albe, K.; Fässler, T. F.; Zobel, M.; Zeier, W. G. Rapid Crystallization and Kinetic Freezing of Site-Disorder in the Lithium Superionic Argyrodite Li6PS5Br. *Chemistry of Materials* **2019**, *31* (24), 10178–10185. https://doi.org/10.1021/acs.chemmater.9b03852.
